# Value-Added Crackers Enriched with Red Onion Skin Anthocyanins Entrapped in Different Combinations of Wall Materials

**DOI:** 10.3390/antiox11061048

**Published:** 2022-05-25

**Authors:** Florina Stoica, Nina Nicoleta Condurache, Georgiana Horincar, Oana Emilia Constantin, Mihaela Turturică, Nicoleta Stănciuc, Iuliana Aprodu, Constantin Croitoru, Gabriela Râpeanu

**Affiliations:** 1 Integrated Center for Research, Expertise and Technological Transfer in Food Industry, Faculty of Food Science and Engineering, Dunarea de Jos University of Galati, 111 Domnească Street, 800201 Galati, Romania; florina.stoica@ugal.ro (F.S.); nina.condurache@ugal.ro (N.N.C.); georgiana.horincar@ugal.ro (G.H.); oana.constantin@ugal.ro (O.E.C.); mihaela.turturica@ugal.ro (M.T.); nicoleta.stanciuc@ugal.ro (N.S.); iuliana.aprodu@ugal.ro (I.A.); 2 Academy of Agricultural and Forestry Sciences, 61 Marasti Blvd, 011464 Bucharest, Romania

**Keywords:** anthocyanins, gelation, red onion skins, antioxidant activities, crackers

## Abstract

The objective of this study was to encapsulate anthocyanins from red onion skins into different biopolymeric matrices as a way to develop powders with multifunctional activities. Two different variants of powders were obtained using a combination of gelation and freeze-drying techniques and characterized by encapsulation efficiency, antioxidant activity, phytochemical content, and color. Stability during storage and the bioavailability of anthocyanins in the in vitro simulated digestion were also examined. Powder V2, with a higher concentration of polysaccharides than V1, allowed a better encapsulation efficiency (90.53 ± 0.29%) and good stability during storage. Both variants had a high content of phytochemicals and antioxidant activity. In vitro investigations proved that an increased polysaccharides concentration offers the best protection for anthocyanins. Thus, a controlled release of the anthocyanins in the intestinal medium was achieved. The powder with the highest encapsulation efficiency was added to crackers, followed by phytochemical characterization to assess its potential added value. The addition of the micro-particles improved the functional characteristics such as antioxidant activity, showing its suitability for the development of bakery products. The attained results may bring implicit benefits to consumers, who can benefit from improved bioactive concentrations in foodstuffs, with significant health benefits.

## 1. Introduction

Onion (*Allium cepa* L.), a vegetable from the *Amaryllidaceae* botanical family, is one of the vital horticultural crops. It is cultivated worldwide in 170 countries and used in various cuisines as a food and medicinal ingredient [1]. In the last few years, the amount of processed onions has expanded, which has driven an increase in onion by-product generation, mainly skin (up to 60%). As a result, the European Union produces around 500,000 tons of onion skins (OS) each year [2]. The onion’s outer dry layers and the first two fleshy layers are usually removed in industrial processing, although these sections are rich in flavonoids [3]. The use of by-products from the food industry is one of the most significant challenges worldwide. The recovered phytochemicals from by-products can be utilized as ingredients to fortify food products, develop functional food products, or in pharmaceutical industries. Regardless of the season, agronomic practices, cultivar type, ripening stage, or storage duration, OS can be a potential source of dietary fibers, alk(en)yl cysteine sulfoxides, fructooligosaccharides, and phenolic compounds [4]. It has been shown that OS possess higher phenolic compounds than the edible parts of the plant [5]. Almost all of the flavonoids found in onions are in the outer layer of the bulb, mainly quercetin and its glycosylated derivatives. Hence, anthocyanins are abundant within the external layer of red onion, mainly cyanidin derivatives, and nearly 60% of them are concentrated within the skin [6]. Red OS could be a source of natural colorants extracted with different techniques and used in foodstuffs as a replacement for synthetic dyestuff [7]. A possible solution for OS valorization may be to utilize it as a good source of food ingredients such as greatly bioavailable flavonoids, fructooligosaccharides, and insoluble dietary fibers [4].

Anthocyanins are water-soluble plant pigments and play a role as natural antioxidants. Furthermore, anthocyanins are non-toxic and may have excellent health benefits in terms of anti-carcinogenic, anti-inflammatory, antioxidant, anti-diabetes, cardioprotective, anti-obesity, neuroprotective, and immunomodulation properties [8]. It is well known that anthocyanins are particularly vulnerable to environmental conditions such as temperature, light, pH, enzymes, and oxygen and are highly susceptible to degradation. Microencapsulation is a suitable technique for protecting anthocyanins, enhancing their bioavailability, improving their stability during storage, and controlling their release in the gastrointestinal tract [9].

The selection of coating material is determined by the ratio of the core material, which acts as an important factor in producing uniform microcapsules with high encapsulation efficiency. Thus, the choice of wall material is fundamental and should be biocompatible and have food-grade status [10]. During the encapsulation of bioactive compounds, the combinations of various types of wall materials might be considered. According to Klein et al. [11], interactions between diverse wall materials might result in complexes with interfacial and amphiphilic features, which improve encapsulation efficiency. Several commonly used wall materials in the food industry are gum arabic (GA), carboxymethyl cellulose (CMC), and soy proteins. CMC is a water-soluble, biocompatible, and biodegradable derivative of cellulose. CMC is used for the encapsulation of active compounds to enhance their bioavailability and stability. Gums have extensive food applications because of their unique functionalities, biocompatibility, non-toxicity, biodegradability, and safety for human consumption [10]. GA is popular for its film-forming capability, low viscosity, aqueous solubility, and emulsification properties [12].

Proteins are an effective carrier for biocompounds, being cheap, biodegradable, and having film-forming, gel-forming, emulsification, and water-holding capacity functionalities. Soy protein isolate (SPI) has remarkable physicochemical functionalities for gel formation, emulsification, water binding, and nutrient protection against oxidation [10]. Soy proteins are recognized as suitable encapsulating materials for various bioactive compounds for their availability, high nutritional value, and lower cost. Plant proteins are more renewable raw materials than animal proteins [13]. The use of a blend of SPI, GA, and CMC could lead to the development of powders with interesting characteristics.

Several techniques have been studied aiming at the microencapsulation of the phenolic compounds of OS, such as physical encapsulation methods (spray and freeze-drying) [14,15].

The method and wall material used significantly affect the final product’s characteristics, including the retention of the active agent, stability, solubility, and antioxidant power. One widely used method for encapsulating heat-sensitive and unstable compounds is the freeze-drying technique, in which water from the product is removed by sublimation. Therefore, this method is a process that maintains the initial functional properties of the encapsulated active compounds. Moreover, freeze-drying shows good properties in process yield, high-quality products, and anthocyanin retention, resulting from the use of low temperatures [16].

The present research aimed to microencapsulate the anthocyanins from red OS extract into a mixture of a complex biopolymeric matrix through the gelation and freeze-drying methods. Due to their ability to generate hydrogels with three-dimensional network complexes, SPI with CMC and GA were utilized as wall materials in various concentrations. Further, the study aimed to comparatively investigate the total anthocyanin content (TAC), total flavonoid content (TFC), total polyphenol content (TPC), anthocyanin entrapping efficiency (EE), and antioxidant activity of the produced powders. The release of anthocyanins from the microcapsules under simulated in vitro gastrointestinal conditions, morphological structure, color, and the storage stability of the bioactives were also studied. In addition, to analyze the powders’ functionality, the one with the highest EE (V2) was incorporated into crackers, which were further characterized for phytochemicals, antioxidant activity, the storage stability of phytochemicals, and sensory attributes. Hence, it is necessary to valorize red OS, rich in active compounds, preventing its dumping through formulations that permit its insertion into food matrices.

## 2. Materials and Methods

### 2.1. Reagents and Chemicals

Soy protein isolate (90% protein content) was purchased from Kemin Industries (Herentals, Belgium). Gum arabic, sodium carboxymethyl cellulose, Folin–Ciocalteu reagent, glacial acetic acid, 2,2-diphenyl-1-picrylhydrazyl (DPPH), HPLC purity methanol, ethanol, 6-hydroxy2,5,7,8 tetramethylchromane-2-carboxylic acid (Trolox), sodium nitrite, Gallic acid, sodium hydroxide, potassium chloride solution, sodium acetate solution, aluminum chloride, sodium carbonate, and sodium bicarbonate were obtained from Sigma Aldrich Steinheim (Darmstadt, Germany). All reagents used in the experiments were of analytical grade.

### 2.2. Red OS Extract Preparation

Red onions were purchased from a local market (Galați, Romania) in July 2021. The outer layers of red onions were collected, cleaned with distilled water, then dried in a conventional oven (Stericell 111, MMM Medcenter, München, Germany) for 2 h at 40 °C, up to a moisture content of 11.0%. The red OS were ground (mean particle diameter of 1 mm), stored at room temperature in a glass jar with an airtight lid, and used for further extraction. The extract was obtained by mixing 50 g of the red OS with 700 mL of 70% ethanol solution acidified with glacial acetic acid (ratio 6:1, *v/v*). The ultrasound-assisted extraction (UAE) method was utilized to extract the bioactive compounds from red OS. The extraction was performed using a sonication bath (MRC. LTD, Holon, Israel) at 25 °C for 30 min with a frequency of 40 kHz. In order to acquire anthocyanin-rich extracts, the process was repeated three times. The supernatants were centrifuged at 6000 rpm for 10 min at 4 °C and collected. The supernatant was concentrated under reduced pressure (AVC 2–18, Christ, Shropshire, UK) at 40 °C and then used for phytochemical characterization and microencapsulation experiments.

### 2.3. Extract Characterization

TAC, TFC, TPC, and DPPH radical scavenging activity were followed in the characterization of red OS extract. 

The determination of TAC was made using the pH-differential method with two reagents: potassium chloride buffer (pH = 1.0) and sodium acetate buffer (pH = 4.5) and reported as mg cyanidin 3-*O*-glucoside (C3G)/g dry weight (DW). The experiment was carried out in triplicate (*n* = 3) with a 0.025 M KCl buffer and a 0.4 M CH**_3_**COONa buffer. A UV-VIS spectrophotometer with data analysis software (Libra S22, Biochrom, UK) was used to measure the absorbances at 520 and 700 nm. The TAC was calculated using equation (1).

TAC (mg C3G/g DW) = (A × MW × DF)**/**ε × L (1)

A = (A520 nm − A700 nm) pH 1.0 − (A520 nm − A700 nm) pH4.5(2)

MW—molecular weight of cyanidin 3-*O*-glucoside (449.2 g/mol); DF—dilution factor; ε—the molar absorptivity of cyanidin 3-*O*-glucoside (26,900 L × mol**^−1^** × cm**^−1^**); and L—the optical path (1 cm).

TFC was evaluated using the aluminum chloride method and the results were reported as mg quercetin equivalent (QE)/g DW. In brief, 0.500 mL of extract was mixed with 2 mL of distilled water and 0.15 mL of sodium nitrite. After 5 min of rest, 0.15 mL of aluminum chloride was added. After another 6 min of rest, 1 mL of sodium hydroxide was added and the absorbance was recorded at 𝜆 = 510 nm. Results were calculated according to the Quercetin calibration curve.

TPC was assessed with the Folin–Ciocalteau (FC) method and the concentrations were reported as mg Gallic acid equivalents (GAE)/g DW. In brief, 0.200 mL of extract was mixed with 15.8 mL of distilled water and 1 mL of FC reagent. After 10 min of rest, 3 mL of sodium carbonate was added and the mixture was kept in the dark for 60 min. Finally, the absorbance was recorded at 𝜆 = 765 nm and results were recorded according to the Gallic acid calibration curve.

Antioxidant activity was measured with the DPPH free radical-scavenging method and reported as mM Trolox Equivalents (TE)/g DW [17]. To measure the in vitro antioxidant activity, a volume of 100 μL of the extract was mixed with 3.9 mL of DPPH stock solution. Afterward, the mixture was kept in darkness at room temperature for 30 min. The absorbance was recorded at 𝜆 = 515 nm and the results were calculated according to a Trolox calibration curve. 

### 2.4. HPLC Investigation of the Anthocyanins from the Extract

In order to identify and quantify the anthocyanin pigments from red OS extract, a chromatographic analysis was conducted using a Thermo Finnigan Surveyor HPLC system coupled to a Diode-Array Detector (Finnigan Surveyor LC, Thermo Scientific, Waltham, MA, USA) and run by the Xcalibur software. The anthocyanins from the extract were analyzed at 520 nm, at an oven temperature of 25 °C, on a Synergi 4u Fusion-RP 80A (150 × 4.6 mm, 4 μm) column. The injection volume was 10 μL for the samples. Before the injection, the samples were first filtered using 0.22 µm syringe filters (Bio Basic Canada Inc., ON, Canada). Separation was accomplished under gradient elution conditions at a flow rate of 1 mL/min and using methanol (solvent A) and 10% formic acid aqueous solution (solvent B) as the mobile phase components. The following gradient was used for the samples: 0–20 min, 9–35% (A); 20–30 min, 35% (A); 30–40 min, 35–50% (A); and 40–55 min, 50–59% (A). The anthocyanin quantification was done using the appropriate standards and data reported in the literature by Donner et al. [18], Sharif et al. [19], and Steimer et al. [20]. 

### 2.5. Microencapsulation of Anthocyanins from Red OS Extract

**Figure 1:**
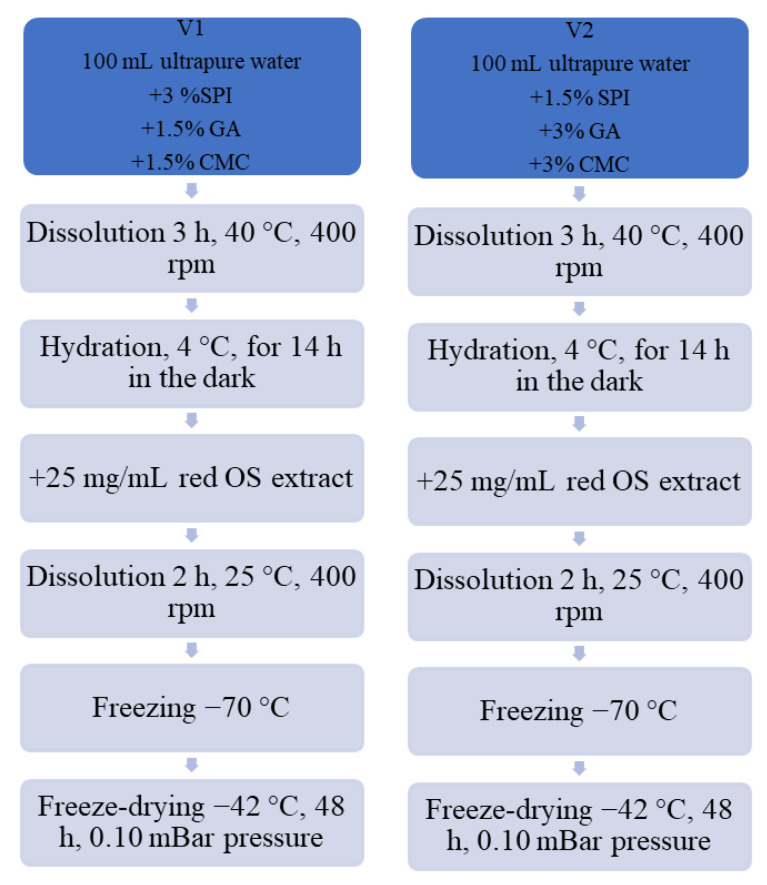
The red OS extract encapsulation diagram, utilizing the gelation and freeze-drying techniques.

SPI–GA–CMC hydrogels containing anthocyanins from red OS were prepared with a modified method described by Serrano-Cruz et al. [21]. Hydrogels are three-dimensional networks of physically or chemically cross-linked hydrophilic polymeric complexes that involve a large amount of water [22]. This study used SPI with CMC and GA as wall materials to obtain two experimental variants of powders. The red OS extract encapsulation diagram, utilizing the gelation and freeze-drying techniques, is provided in Figure 1. Specifically, for variant V1, 3% SPI, 1.5% CMC, and 1.5% GA, were mixed and dissolved in ultrapure water at approximately 40 °C for 3 h and 400 rpm on a magnetic stirrer (IKA RCT Basic, Staufen Germany). Variant V2 was acquired by mixing 1.5% SPI with 3% CMC and 3% GA in ultrapure water and hydrated for 3 h at 40 °C and 400 rpm on a heating magnetic stirrer. After that, to ensure total hydration, the resulting solutions were stored at 4 °C overnight. Next, the aforementioned red OS anthocyanin extract (25 mg/mL) was added to each solution containing the wall materials. The solutions were mixed under strong magnetic stirring for 2 h at 25 °C and 400 rpm, until complete homogenization. The pH values were measured at 3.5. The dispersions were frozen at −20 °C and then were placed in a freeze-dryer (CHRIST Alpha 1–4 LD plus, Osterode am Harz Germany) and dried at −42 °C under a pressure of 0.10 mBar for 48 h. Powders were packed in plastic tubes, sealed, and stored in the absence of light at 4 °C until analysis.

### 2.6. Powder Characterization

#### 2.6.1. Encapsulation Efficiency

The resulting powders were investigated in terms of TAC, TFC, TPC, and antioxidant activity. The surface anthocyanin content (SAC) and total anthocyanin content (TAC) of the powders were used to calculate the EE [23]. 

A weight of 200 mg from each powder was mixed with 5 mL of methanol, acetic acid, and water (25:4:21, *v*/*v*/*v*) for TAC. The mixtures were vortexed (1 min) and sonicated to break the microcapsules (DCG-80H, MRC Scientific Instruments Ltd., Holon, Israel) for 20 min at 40 ± 1.0 °C. The blends were further centrifuged at 14,000 rpm for 10 min at 4 °C.

SAC was determined using 200 mg of each powder with 5 mL of ethanol:methanol mixture (1:1, *v*/*v*) and vortexed (BV 1000 Vortex mixer Benchmark Scientific Inc., Edison, NJ, USA) for 1 min. Afterward, samples were centrifuged for 10 min at 14,000 rpm and 4 °C. TAC and SAC of the supernatants were spectrophotometrically determined utilizing the pH-differential method. The EE of anthocyanins was calculated using Equation (3), as follows:

EE (%) = (TAC – SAC)/TAC × 100(3)

#### 2.6.2. Color Evaluation of Powders

The color attributes of the powders were measured using a MINOLTA Chroma Meter model CR-410 (Konica Minolta, Osaka, Japan) with a CIE Lab scale. The method reads the Chroma parameters by inserting the probe into the powders. The results of color measurements were expressed as L* indicating the lightness (black: L* = 0 and white: L* = 100), a* (red (>0) to green (<0) color), and b* (yellow (>0) to blue (<0) color). The CIELAB color parameters were acquired in triplicate following equipment calibration against a white plate. Hue angle (hue angle = 360 + arctan(b*/a*) for quadrant IV (+a*, −b*), which indicates the color of the powders (0° or 360° = red color, 90° = yellow color, 180° = green color, and 270° = blue color), and the Chroma (Chroma **=** =$\sqrt{{\left(a^{*}\right)}^{2}+{\left(b^{*}\right)}^{2}}$), which indicates the color’s purity or saturation, were also calculated [24].

#### 2.6.3. Storage Stability Studies

The powders were filled in plastic tubes and kept at 5 °C in the dark. They were analyzed for their bioactive contents and antioxidant activities, as previously described, during 28 days of storage.

#### 2.6.4. Scanning Electron Microscopy (SEM) Analysis

The morphological examination of the fine powder coded V2 was performed by SEM, using the Quanta 200 scanning electron microscope (Philips FEI). V2 powder was chosen due to its highest EE.

The sample was analyzed at a pressure of 60 Pa, in low vacuum mode, while the acceleration voltage of the electrons in the electronic cannon was 15 kV. The powder was fixed on an aluminum tube with the help of a carbon double adhesive tape. The samples were coated with a 7 nm thick layer of gold using an SPI-Module Sputter Coater (SPI Supplies, West Chester, PA, USA) with an intensity of 18 mA to increase the conductivity. SEM images were collected at different magnifications between 200 and 10.000X to highlight the microstructures or morphological changes in the powder.

#### 2.6.5. Determination of the In Vitro Release of Anthocyanins from Microcapsules

The release of anthocyanins from microcapsules under simulated in vitro digestive conditions was investigated as described by Minekus et al. [25]. Both powders were subjected to gastrointestinal digestion. Simulated gastric fluid (SGF) containing porcine pepsin (40 mg/mL in 0.1 M HCl, pH = 3.0) was used to simulate gastric digestion. Simulated intestinal fluid (SIF) containing pancreatin from porcine pancreas (2 mg/mL in 0.9 M sodium bicarbonate, pH = 7) was used to simulate intestinal digestion. The samples were shaken for 2 h per digestion in a shaker (Medline Scientific, Chalgrove, Oxon, UK) at 150 rpm and 37 °C during the experiment. Every 30 min, 0.2 mL of each sample’s mixture was collected for TAC quantification in order to determine the percentage of anthocyanins released from the experimental powders. Each collected sample was centrifuged before TAC determination at 14,000 rpm for 10 min at 4 °C.

### 2.7. Preparation and Characterization of Value-Added Crackers

In order to obtain value-added crackers, V2 powder was used in two different ratios (1% and 3%) to test its functionality. The control sample (Control) consisted of crackers without powder added. The crackers were prepared with the following ingredients: 43 g wholemeal rice flour (21.54%), 23 g buckwheat flour (11.52%), 20 g butter (10.02%), 5 g olive oil (2.5%), 10 g egg yolk (5.01%), 35 g water (17.53%) 60 g pressed cheese (30.6%), 1.4 g caraway seeds (0.7%), 0.2 g black pepper (0.1%), and 2 g of salt (1%). First, butter, olive oil, egg yolk, and salt were mixed for 3 min at 120 rpm. Then, the other ingredients were added and mixed at high speed for another 10 min. The two types of flour were added in two separate steps, each followed by 1 min of mixing at level 1. The obtained dough was cooled at 4 °C for 20 min. The next step was cutting the cracker dough into various shapes. The crackers’ shapes were then baked at 160 °C for 15 min. After baking, the crackers were allowed to cool at room temperature, packed in zipped polyethylene bags, and stored in the dark at room temperature until further analysis. Based on the powder concentration (1% and 3%), the samples were coded as C1 and C2. The stability of value-added crackers’ phytochemicals and radical scavenging activity were tested during 28 days of storage at 25 °C.

#### Sensory Evaluation of Value-Added Crackers

Ten untrained tasters performed a sensory evaluation of the crackers. The participants were consumers of biscuits (appetizers) and pastries. They were given information about the study’s overall goal as well as the required protocols for managing personal data. The panelists were asked to evaluate the appearance, section appearance, firmness, chewability, color, consistency, taste, smell, aroma, aftertaste, and general acceptability of the value-added crackers utilizing a 9-point hedonic scale (1 = extremely dislike; 9 = extremely like). The samples were given three random digit codes and served at room temperature (23–25 °C), under white light, and with 45–48% relative air humidity. The order in which the samples were presented was randomized. After each sample evaluation, panelists were advised to rinse their mouths with water.

### 2.8. Statistical Analysis

All analyses were performed in triplicate, and the results were expressed as mean ± standard deviation. Experiments were submitted to one-way analysis of variance (ANOVA) using Minitab 19 (Minitab Inc., PA, USA), followed by Tukey’s test for comparison of means, considering a 5% significance level (*p* < 0.05).

## 3. Results and Discussion

### 3.1. Red OS Phytochemical Characterization

The UAE method was performed to extract bioactives from red OS, and 70% ethanol acidified with glacial acetic acid (ratio 6:1, *v/v*) was chosen as the solvent mixture. The bioactive compound content and the antioxidant activity of the red OS extract were determined. In our study, the extract recorded a TAC of 1.67 ± 0.14 mg C3G/g DW. The TFC was 194.60 ± 0.74 mg QE/g DW, whereas the TPC was 121.30 ± 0.98 mg GAE/g DW. The extract showed high antioxidant activity of 47.32 ± 0.15 mM TE/g DW, with a corresponding inhibition of 76.20 ± 0.24%.

Katsampa et al. [26] reported that 90% aqueous glycerol extracts of red OS produced higher TAC (1.87 ± 0.39 mg C3G/g DW) than the present study. However, they reported a lower TPC (61.47 ± 14.19 mg GAE/DW) using a 90/1 solvent/solid ratio at 45 °C for 60 min of sonication. Sagar et al. [27] analyzed the phytochemical content of the red OS of the *Pusa Red* cultivar and found a higher TPC of 251.71 ± 1.21 mg GAE/g DW than our study and a lower TFC of 89.62 ± 0.70 mg QE/g DW. In addition, the DPPH scavenging activity was 69.97 ± 0.12% after the UAE extraction of bioactives from red OS using methanol as an extraction solvent. Bordin Viera et al. [28] reported a lower TAC of dried red OS ranging from 0.82 to 4.31 mg C3G/g DW compared to ours. Moreover, the authors stated that the TPC in the UAE of red OS ranged from 117.50 to 822.87 mg GAE/g DW, with greater values observed in the continuous mode (130 and 750 W) and pulsed mode (750 W) with increasing solvent concentration (60–80%).

The phytochemical content of extracts can vary with the cultivar, different extraction conditions (e.g., type of solvent, temperature, pH, and light intensity), agronomic factors, and the measurement methods applied. Up to now, it can be concluded that red OS extracts are a good source of phytochemicals with antioxidant activity and may be utilized as ingredients in food products, contributing to the reduction in agro-industrial residues.

### 3.2. HPLC Investigation of Phenolic Compounds from the Extract

**Figure 2:**
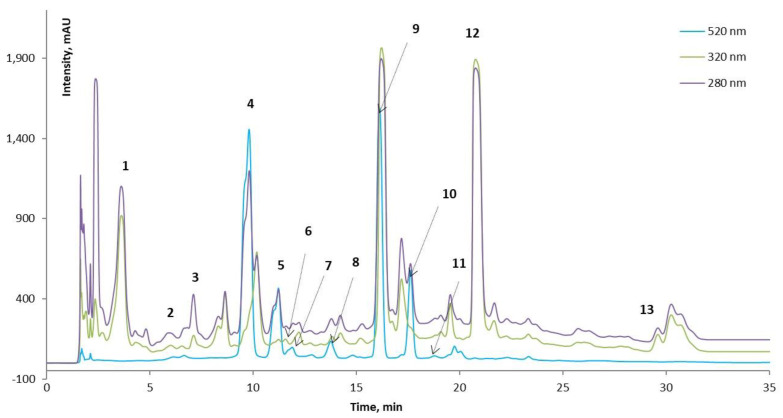
HPLC chromatograms of phenolic and polyphenolic compounds quantified in the extracts of red OS: peak 1-catechin, peak 2-chlorogenic acid, peak 3-vanillic acid, peak 4-cyanidin 3-laminaribioside, peak 5-cyanidin 3-(3″-malonylglucoside), peak 6-ferulic acid, peak 7-peonidin 3-glucoside, peak 8-cyanidin 3-(6″-malonylglucoside), peak 9-cyanidin 3-(6″-malonyl-laminaribioside), peak 10-peonidin 3-malonylglucoside, peak 11-cyanidin 3-dimalonylaminaribioside, peak 12-quercetin, and peak 13-kaempferol.

The typical HPLC chromatogram of phenolic compounds from the extract is given in Figure 2. The study used detection wavelengths of 520 nm, 280 nm, and 320 nm. The identification of the phenolic compounds was achieved based on the retention time (RT) and by comparison to the available standards (chlorogenic, acid, ferulic acid, catechin, quercetin, kaempferol), as well as the existing data in the specialized literature. The chromatograms at 320 nm and 280 nm indicated the presence of many compounds, of which only six could be tentatively identified, such as peak 1—catechin, peak 2—chlorogenic acid, peak 3—vanillic acid, peak 6—ferulic acid, 12—quercetin, and peak 13—kaempferol. Among them, the main compound identified was quercetin. The high levels of quercetin in the outer layers of onion bulbs can be explained by sunlight exposure [5]. Sagar et al. [27] obtained similar results in a study on fifteen Indian onion cultivars. In this case, the flavonoids identified were quercetin, quercetin 3-β-D-glucoside, luteolin, and kaempferol. Zhang et al. [29] also presented quercetin and quercetin 3-glycoside as the main flavonols in onions.

Figure 2 illustrates that seven anthocyanins were separated at 520 nm: peak 4—cyanidin 3-laminaribioside, peak 5—cyanidin 3-(3″-malonylglucoside), peak 7—peonidin 3-glucoside, peak 8—cyanidin 3-(6″-malonylglucoside), peak 9—cyanidin 3-(6″-malonyl-laminaribioside), peak 10—peonidin 3-malonylglucoside, and peak 11—cyanidin 3-dimalonylaminaribioside. Our results are in agreement with other studies by Rice-Evans et al. [30], Donner et al. [18], and Sharif et al. [19]. In another study, Gennaro et al. [31] identified delphinidin or petunidin derivatives at very low levels regarding cyanidin derivatives. Donner et al. [18] identified eight anthocyanin compounds based on their HPLC chromatogram, and thereby, the principal pigment was cyanidin 3-malonylglucoside (51.4 mg/100 g DW). Other authors, such as Celano et al. [32], detected in “*Rossa di Tropea*” OS extracts four cyanidin derivatives (cyanidin 3-glucoside, cyanidin 3-laminaribioside, cyanidin 3-malonilglucoside, and cyanidin 3-malonillaminaribioside). Cyanidin 3-glucoside and cyanidin 3-laminaribioside are the main anthocyanins identified in red onions. The authors highlighted the content of 365.2 ± 4.5 mg/100 g DW for cyanidin 3-glucoside and 34.5 ± 2.3 mg/100 g DW for cyanidin 3-malonylglucoside, respectively. Rice-Evans et al. [30] discovered delphinidin 3-laminaribioside in OS and cyanidin 3-malonylglucoside and cyanidin 3-malonyllaminaribioside. In another study, four main anthocyanin aglycones in red onion were identified, such as delphinidin 3,5-diglycosides, cyanidin 3,5-diglycosides, and cyanidin 3-glycosides, and cyanidin 3-(6″-malonyl)-glucopyranoside [29].

### 3.3. Powders Characterization and Encapsulation Efficiency

The encapsulation technique must present a high retention rate of the active compound to effectively reach its site of action in high amounts. EE is defined as the percentage of the active ingredient that is successfully captured in the carrier material of the microcapsule. The efficiency of encapsulation is influenced by the properties of shell and core materials, drying parameters, and the colloidal microsystem characteristics [33]. Table 1 summarizes the results of the phytochemical characterization of powders. The encapsulating wall materials used had a significant impact on the EE of the samples. Thus, V2 powder with a higher concentration of polysaccharides had the highest anthocyanin EE (*p* < 0.05). Hence, the EE increased significantly with the concentration of carbohydrate polymers, ranging from 82.46 ± 0.92% for V1 to 93.19 ± 1.12% for V2 (*p* < 0.05). This difference between the two encapsulation efficiencies could be explained by the polysaccharides’ disruptive effect on the protein/polyphenol complex in a matrix during encapsulation, as stated by Thongkaew et al. [34]. Bourvellec and Renard [35] also explained that this phenomenon appears due to the ability of polysaccharides to encapsulate phenolic compounds, competing with the binding to proteins. They also stated that ionic polysaccharides such as pectin are known as effective inhibitors of polyphenol/protein complexation.

Our reported data are in agreement with other studies. In the study of Mansour et al. [36], red raspberry anthocyanins were encapsulated in 2.5% *(w/v)* of SPI and 2.5% *(w/v)* of GA and led to the highest efficiency of 98.87%. Santana et al. [37] studied the microencapsulation of jussara pulp (*Euterpe edulis*) by spray-drying. They reported EE values varying from 80.27 to 99.50% and 80.33 to 99.33% for mixtures with GA, modified starch, whey protein concentrate and GA, modified starch, and SPI. The combination of carboxymethyl starch (CMS) and xanthan gum (XG) produced an EE for blueberry anthocyanins of over 96% in the research of Cai et al. [38]. Furthermore, Pereira Souza et al. [39] established that the anthocyanin retention and antioxidant activity of *Jaboticaba* pomace microencapsulated in SPI combined with maltodextrin were more effective than maltodextrin, maltodextrin-pectin, and maltodextrin-pectin-SPI carriers. Cilek et al. [40] showed that the freeze-drying encapsulation of phenolic compounds from cherry pomace using MD and GA at different ratios (10:0, 8:2, and 6:4) had an EE increasing with the ratio of GA (78–92%). The powder’s EE difference may be attributed to the different properties of the shell materials and their response during the drying process. 

In our research, the combination of red OS powders obtained via gelation and freeze-drying exhibited different phytochemical profiles (Table 1). The powders showed that TAC, TPC, TFC, and antioxidant activity values were significantly greater in V2 than in V1 (*p* < 0.05). Higher polysaccharide concentrations than protein concentrations as carrier materials permitted the retention of higher amounts of phytochemical compounds from red OS extract. Our findings reached more satisfactory values for the phytochemical contents of the powders than those reported by Condurache et al. [41], who encapsulated bioactive components from eggplant peel extract using various combinations of sodium carboxymethyl cellulose (CMCNa), pectin (P), and whey protein isolate (WPI). They reported a TAC of 0.095 ± 0.01 mg delphinidin 3-*O*-glucoside/g DW, a TFC of 1.64 ± 0.14 mg catechin equivalent/g DW, a TPC of 7.22 ± 0.18 mg GAE/g DW, and antioxidant activity of 36.60 ± 0.83 mM TE/g DW for the powder with higher CMCNa and P concentrations. 

Therefore, the encapsulation matrix containing CMC, GA, and SPI effectively formed colloidal particles, successfully encapsulating the red OS extract’s bioactive compounds.

### 3.4. Color Evaluation of Powders

The results of the color measurement (L*, a*, b*, and parameters Chroma and hue angle) after obtaining the microcapsules and after 4 weeks of storage at 5 °C are revealed in Table 2. The L* values, which show the lightness of the samples, ranged between 21.80 ± 0.30 and 22.56 ± 0.18 for V1 and V2 powders. Both powders’ lightness decreased significantly (*p* < 0.05) after 28 days of storage. The two powder samples had a high value of color parameter a*, with the highest value for the V2 powder, which can be attributed to the higher anthocyanins content, as stated by Jimenez-Aguilar et al. [42]. The positive value of the parameter a* shows a tendency to originate the red color.

The blue-to-yellow intensity is represented by the parameter b*, with a negative value indicating a trend towards blue shades in powders. The b* values increased significantly (*p* < 0.05) after storage at 5 °C for 4 weeks for all samples. Increased b* values can be attributed to the loss of co-pigmentation effects and the formation of pyranoanthocyanins, according to Tsali and Goula [43].

Chroma followed the trend of the parameter a*, suggesting that the red color was the most expressive in deciding the color of the powders. The hue angle was positioned in the first quadrant in the color solid, indicating the redness of all samples, since 0 and 360 angles are attributed to red. The hue angle exhibited a slight decrease for all tested samples during storage. Parameter a* and Chroma increased significantly (*p* < 0.05) after 4 weeks of storage, with V2 having the highest degree of color saturation, which is a desirable attribute. Thus, the samples reduced in lightness and their color became darker after storage.

According to the results obtained for the values of a* and b*, all data were placed in the fourth quadrant (+a*, −b*), suggesting a tendency toward blue and red, which is characteristic of anthocyanins. De Souza et al. [44] reported a similar result for the *Bordo* grape skin extract encapsulated by spray-drying with 10% maltodextrin.

### 3.5. Storage Stability Studies of the Powders

**Figure 3:**
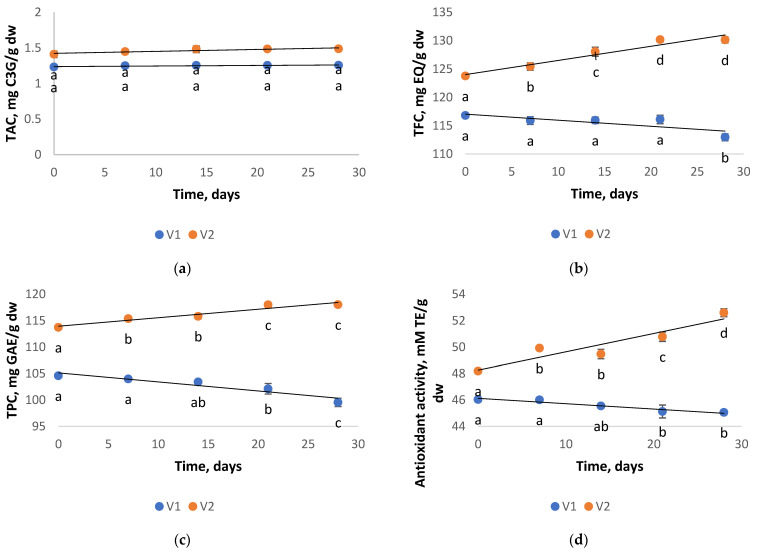
The TAC (**a**), TFC (**b**), TPC (**c**), and antioxidant activity (**d**) stability of the V1 and V2 powders after 28 days of storage at 5 °C. For each tested phytochemical and storage time of the powder variant, values sharing different letters indicate a significant difference, *p* < 0.05.

The storage stability of powders concerning polyphenol, flavonoid, anthocyanin contents, and antioxidant activity is presented in Figure 3. The stability of powders was evaluated during 4 weeks of storage at 5 °C. The TAC of both powders did not significantly change during the storage period (*p* > 0.05). Regarding the TFC values of the V1 powder, a decrease of nearly 3% was noticed over the tested storage period. An improved TFC stability was registered for the V2 sample encapsulated with higher polysaccharide concentrations; a slight increase of almost 5% was noticed after 28 days of storage. Figure 3 reveals a significant 5% decrease in the TPC obtained for the V1 powder after 4 weeks of storage, whereas in the case of V2 powder, an approximately 4% increase in TPC was observed. Moreover, the V2 powder antioxidant activity increased by 8% until the end of the storage period, while V1 presented a 2% decrease in the antioxidant activity. 

The increase in the content of flavonoids, polyphenols, and implicit in the antioxidant activity of powder V2 may be due to the possible hydrolysis of conjugated polyphenols, as stated by various authors (Zhang et al. [45]). During storage, the phenolic compounds’ structure may be changed as some compounds could be degraded, while other new phenolics could be formed, increasing the TPC. Ribeiro et al. [46], on the other hand, stated that the oxidation and degradation phenomena that happen during storage might lead to the formation of false-positive values in the release profiles. The degradation reactions could lead to the formation of sub-products. These products could be quantified during the analyses since the FC method and the analysis of the release profiles by the UV/Vis spectrometry tests are quantitative but not specific, counting the polyphenols and other compounds that remain in the sample.

In the study of Cai et al. [38], the highest blueberry anthocyanins retention was observed for a combination of CMS and XG, (CMS/XG:150/1), which was 90.47% at 5 °C and 76.11% at 37 °C after 30 days. According to Sanchez et al. [47], after 60 days of storage, 90% of the anthocyanin from cherry juice could be retained in the samples freeze-dried with GA and maltodextrin. Çam et al. [48] stated that the TPC from pomegranate skin encapsulated in maltodextrin exhibited a change from 129.1 to 119.3 mg GAE/g sample within 60 days of microcapsules stored at 4 °C. The retention of antioxidant activity up to 88% has been reported by Brauch et al. [49] in maqui juice freeze-dried with maltodextrin. Likewise, it has been found that a spray-dried black currant extract encapsulated with inulin can maintain practically unmodified antioxidant activity for 12 months of storage at 8 °C, although at 25 °C, only small changes occur [50].

From Figure 3, a minor phytochemical content variation in time can be seen. However, we could deduce that the utilized combination of a greater concentration of CMC and GA effectively encapsulated the anthocyanins, offering them good stability. The values obtained in our findings suggest that the mixture with higher concentrations of polysaccharide and lower protein as wall materials enhanced the encapsulation of anthocyanins from red OS extract and their stability during storage. Therefore, the freeze-drying of red OS extract using a mixture of lower protein concentrations and higher polysaccharides concentrations resulted in more stable powders, abundant in anthocyanins and with high antioxidant activity, which can be utilized as a functional ingredient in a variety of food products.

### 3.6. Morphological Structure of the V2 Powder

**Figure 4:**
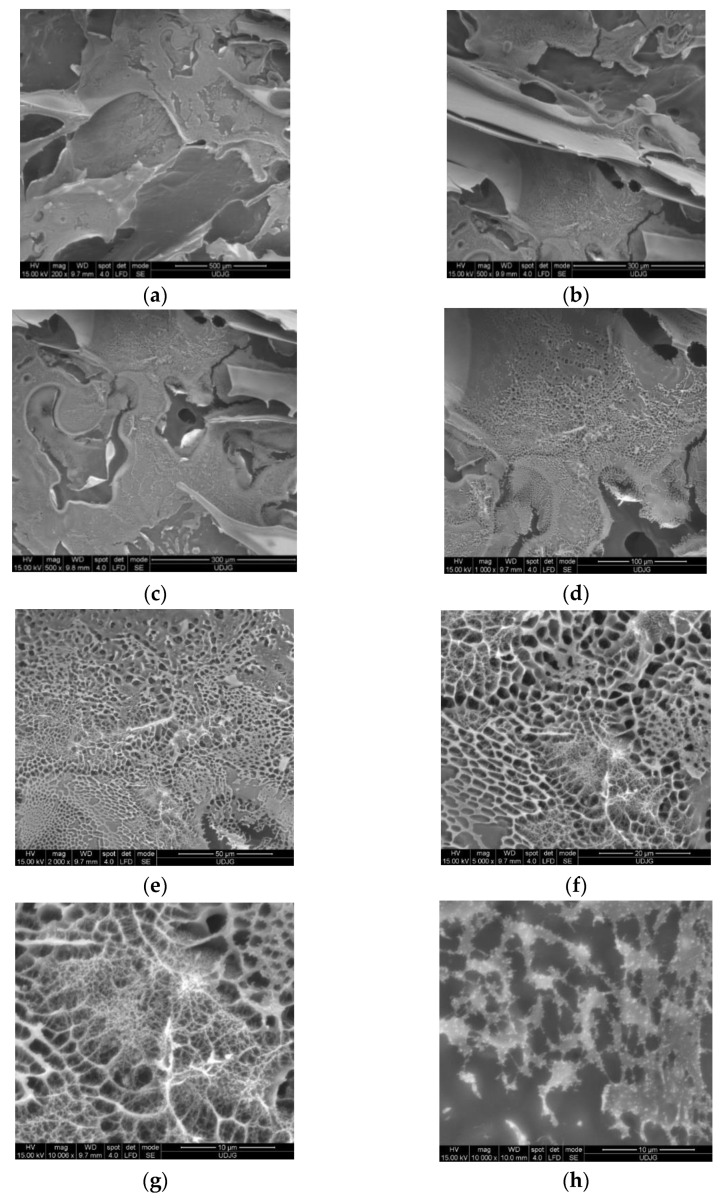
SEM micrographs of the V2 powder at magnification of 200× (**a**), 500× (**b**,**c**), 1000× (**d**), 2000× (**e**), 5000× (**f**) and 10000× (**g**,**h**).

The structural properties of V2 microcapsules were analyzed by SEM spectroscopy. The SEM analysis was used to remark the external morphology of the V2 microparticles obtained by the microencapsulation of red OS extract in 1.5% SPI with 3% CMC and 3% GA. The micrographs were attended at different magnification due to the sharpness and size of the microcapsules generated by the freeze-drying method. Generally, it was noted that the powder had an irregular structure with different particle sizes and shapes. The SEM images (Figure 4a–h) revealed the formation of aggregates made of plates apparently without structure. When increasing the magnitude (500–1000×, c–d), a structure of the plates could be noticed in the form of an irregular network. The formation of the network is associated with the coating agent’s composition and may be given by the long carboxymethyl cellulose fibers. Variations in the surface morphology of freeze-dried powder were noticed, which could be attributed to the coating agents’ properties. In Figure 4b, at 500×, a lamellar structure for V2 powder particles can be remarked. In Figure 4f,g (5000×, 10000×), it can be noticed that inside the meshes of the network mentioned above are other developed networks with a thinner structure. In addition, this structure is not uniform and is not developed on all the meshes of the larger network. These appearances may be explained by adding the wall materials such as soy protein and the GA and by the interactions between polyphenols and soy protein, as stated by Bandyopadhyay et al. [51].

In the SEM image 4h, which represents the detail of the thinner network, it can be seen that there are many bright spots with dimensions lower than 1 µm, which can be associated with vesicles (protuberances) on the surface formed by the thinner network. According to the study of Laokuldilok and Kanha [52], the SEM analysis of freeze-dried anthocyanin particles from black glutinous rice bran fraction resulted in irregular agglomerates and a thin, porous, sheet-like material. Roughness in microparticles is typically caused by particle shrinkage after a large moisture loss and subsequent cooling. The quick sublimation of frozen water from the matrix may have resulted in the development of cavities where ice crystals previously existed, as Al-Maqtari et al. [53] explained.

### 3.7. In Vitro Release of Anthocyanins from the Microcapsule

**Figure 5:**
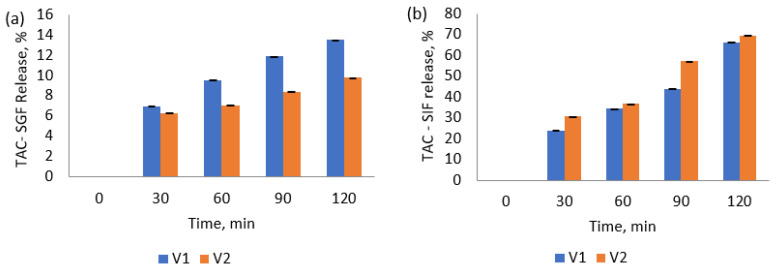
Anthocyanin release from the microcapsule powders during the simulated in vitro gastric (**a**) and intestinal (**b**) digestion.

The changes in the anthocyanin concentrations of the powders were monitored during in vitro simulated gastrointestinal fluids for 4 h (Figure 5). The results obtained displayed that the protective coating of biopolymeric wall materials effectively controlled the release of encapsulated anthocyanins. Thus, biopolymeric matrices had a protective effect on anthocyanins in the gastric phase but a releasing effect in the intestinal phase.

Anthocyanins in the experimental powders were stable during the incubation in the SGF for 2 h (Figure 5a). The powders slightly increased the anthocyanin content during gastric digestion, with approximatively 13.5% for V1 and 10% for V2 after 120 min. The results suggested that the microcapsules coated by the ternary matrices were resistant to the gastric environment of the stomach, and they protect the anthocyanins during passage through the human stomach. In SIF (Figure 5b), the data exhibited that the anthocyanins’ release after 120 min of digestion reached a maximum of 66% for V1 and 70% for V2. Results showed a controlled release of anthocyanins under intestinal conditions, meaning that a significant amount remained in the microcapsules. 

Our results are in corroboration with other studies. Mansour et al. [36] encapsulated the anthocyanins from red raspberry extract with SPI and GA. The authors revealed that the SPI and GA combination presented good release behavior for anthocyanins under simulated gastric and intestinal digestion compared to non-encapsulated anthocyanin extract. Huang and Zhou [54] found that using GA to encapsulate black rice anthocyanins helped delay the release of anthocyanins during in vitro simulated gastrointestinal digestion. The bioaccessibility of blackberry anthocyanins with β-cyclodextrin as encapsulated wall material was 11% higher than non-encapsulated anthocyanins during in vitro simulated digestion [55]. 

This study showed that CMC, GA, and SPI effectively prevent the release of anthocyanin in gastric conditions, which increases their bioavailability by minimizing the chemical degradation of anthocyanins in the gut environment. Thus, a higher anthocyanin protective effect can be noticed by the higher concentration of polysaccharides (V2). 

### 3.8. Characterization of Value-Added Crackers

In order to check the V2 powder as a multifunctional food ingredient, different percentages of 1% (C1) and 3% (C2) were added to a recipe for crackers. The formulated food products’ bioactive stability (TAC, TFC, TPC, and antioxidant activity) was tested after 28 days of storage at 25 °C. Table 3 shows the phytochemical content of the experimental food products and stability during 28 days of storage. The addition of the microcapsules into the biscuits’ formulation resulted in increases in TPC, TFC, and TAC levels. The presence of phenolic compounds in the control sample was related to the main ingredients used to make the crackers, lacking in anthocyanins. In vitro, the antioxidant activity of the enriched biscuits was positively influenced by bioactive compounds extracted from red OS, and they displayed an antioxidant activity higher than that of the control crackers. 

As expected, there is a significant increase in the concentration of phytochemicals by increasing the powder concentration in the developed crackers (*p* < 0.05). Thus, the cracker variants presented a TAC with values between 27.57 ± 0.24 μg C3G/g DW and 39.18 ± 0.65 μg C3G/g DW. The antioxidant activity in the added-value crackers was higher when compared to the control, with 58% in C1 and 64% in C2, respectively. The data presented in Table 3 confirm the added value of the formulated crackers with microencapsulated red OS extract by increasing the content of anthocyanins and antioxidant activity, respectively. After 28 days of storage, a significant decrease (*p* < 0.05) was found in the anthocyanin contents of the formulated crackers, of 24% and 17% for C1 and C2, respectively. Moreover, a significant decrease during the entire storage period was observed in the polyphenol contents of the two formulated crackers, while the flavonoid contents of cracker variants remained constant. From Table 3, a significant decrease of 8%, and 6% (*p* < 0.05) in the antioxidant activity of the C1 and C2 developed crackers over the storage time can be remarked. 

Papillo et al. [56] obtained enriched biscuits with *Artemide* black rice extract microencapsulated in maltodextrin and GA and showed a higher anthocyanins content (32–20 mg/g biscuit) and antioxidant capacity compared to a control biscuit. Kaderides et al. [57] used pomegranate peel phenolic extract encapsulated in maltodextrin added to cookies at 0.5% (*w/w*) concentration. However, adding phenolic compounds to enriched cookies substantially affected antioxidant activity retention. Thus, the antioxidant activity of the cookies remained high and was significantly higher than that of the control cookies after 21 days of storage. Tumbas Šaponjac et al. [58] reported that polyphenols had shown an increase of 42% in cookies enriched with 10% encapsulated powder from sour cherry pomace extract in soy proteins, while anthocyanins (64.33%) and antioxidant activity (24.30%) decreased after 4 months storage at 25 °C. The replacement of flour with encapsulated powder improved the functional value of the cookies and enhanced the storage stability.

### 3.9. Value-Added Crackers Sensorial Analysis

**Figure 6:**
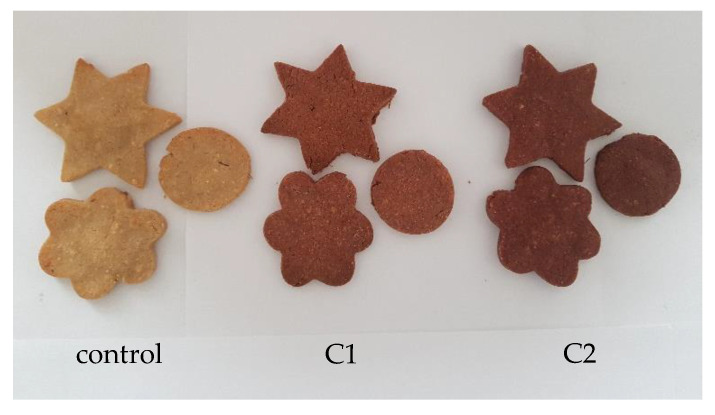
Crackers with different percentages of encapsulated red OS extract: control, 1% (C1), and 3% (C2).

The sensory evaluation of the formulated biscuits was realized using a nine-point hedonic scale. The sensorial analysis was performed following the sensorial characteristics such as appearance, section appearance, firmness, chewability, color, consistency, taste, smell, aroma, aftertaste, and general acceptability. With the increase in powder concentration, the crackers appeared more reddish-brown in color due to the presence of anthocyanins (Figure 6).

**Figure 7:**
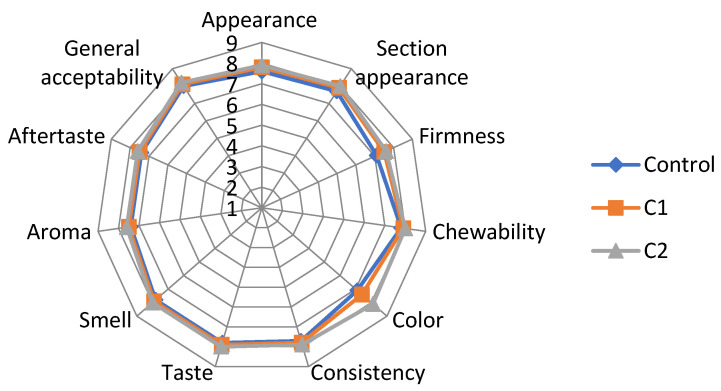
Comparative diagram of the sensory attributes specific to crackers: Control = crackers without powder addition; C1 and C2 = crackers with, respectively, 1 and 3% powder of red OS extract.

The average scores obtained from the sensory evaluation are displayed in Figure 7. Sample C2 with 3% red onion encapsulated extract obtained the highest scores for all 11 attributes. Even though the control sample received the lowest total scores, in some attributes, such as firmness, aroma, and aftertaste, it was just as well appreciated as the other samples. The biscuits had regular-shaped whole layers with a matte, semi-gloss surface. In the section, they were well-baked with uniform layers without gaps. The surface of biscuits with microcapsules had less sheen and a rough surface. The taste was pleasantly salty, with an aroma of caraway. The likeness score for taste indicated that the panelists liked the taste of the crackers. The taste of the C1 and C2 biscuits with V2 powder was comparable to the control. 

The aroma of the crackers was neither liked nor disliked by the panelists. In general, the aroma scores were still above the acceptable level, with scores of >7. The tasters noted that the samples’ colors ranged from golden-yellow to reddish-brown, with C2 being the most reddish-brown. The biscuits with red OS powder were found to have a balanced flavor, taste, and aftertaste. Moreover, the biscuits’ crunchy, tender, and non-crumbly consistency was appreciated. The panel participants positively evaluated all tested samples, with no perceived red OS flavor. The results of the sensory evaluation suggest that crackers in which 3% of powder was added showed the highest value of “general acceptability”.

## 4. Conclusions

The results highlighted that the microencapsulated red OS extract is an important source of bioactive compounds with high antioxidant activity. In this study, we could efficiently encapsulate red OS extract in an IPS–CMC–GA matrix by the combination of gelation and freeze-drying methods with an entrapping efficiency of 82.46 ± 0.92% for V1 and 93.19 ± 1.12% for V2, respectively. The biopolymer combinations used with the highest concentration of polysaccharides (V2) as carrier agents resulted in better maintenance of the anthocyanins and antioxidant potential of red OS powder during storage for 28 days at 5 °C. The SEM analysis revealed that the V2 powder presented vesicles (protuberances) on the developed irregular network surface. The microcapsules showed less L* value and a high a* value according to their anthocyanin contents, establishing their unique potential to be used as colorants. In addition, the simulated digestion indicated that anthocyanins were effectively protected by the selected matrices against in vitro gastric digestion and were delivered in the simulated intestinal phase. The sensory tests revealed that the developed crackers with added powder might be potentially well accepted among consumers, having fairly acceptable quality attributes. The results of this work display that red OS powder could be a promising alternative for the food industry to provide anthocyanin-enriched crackers. Thus, adding red OS powder to crackers improves certain nutritional properties compared with the control sample. This work concedes the possibility of using the encapsulated anthocyanins from red OS as a new functional ingredient that can be used as a natural red colorant and opens up new prospects of using them in anthocyanin-enriched food products with potential health benefits.

## Figures and Tables

**Table 1:** Characterization of the phytochemicals of red OS powders.

Phytochemical Content	TAC, mg C3G/g DW	TFC, mg QE/g DW	TPC, mg GAE/g DW	Antioxidant Activity, mM TE/g DW	Encapsulation Efficiency, EE %
V1	1.23 ± 0.04 ^b^	104.60 ± 0.20 ^b^	116.79 ± 0.15 ^b^	46.04 ± 0.17 ^b^	82.46 ± 0.92 ^b^
V2	1.41 ± 0.02 ^a^	113.72 ± 0.19 ^a^	123.75 ± 0.24 ^a^	48.19 ± 0.16 ^a^	93.19 ± 1.12 ^a^

Different letters in the same column suggest statistically significant differences (*p* < 0.05).

**Table 2:** Color parameters (L*, a*, b*, hue angle, and Chroma) of the powders.

Sample	Storage Time (Days)	L*	a*	b*	Hue Angle	Chroma
V1	0	21.80 ± 0.30 ^aB^	17.29 ± 0.02 ^bB^	−0.93 ± 0.21 ^bA^	359.95 ± 0.01 ^aB^	17.31 ± 0.03 ^bB^
	28	16.73 ± 0.11 ^bB^	18.34 ± 0.12 ^aB^	−0.86 ± 0.01 ^aA^	359.94 ± 0.01 ^aB^	18.36 ± 0.06 ^aB^
V2	0	22.56 ± 0.18 ^aA^	20.84 ± 0.07 ^bA^	−0.78 ± 0.07 ^bB^	360.04 ± 0.01 ^aA^	20.85 ± 0.07 ^bA^
	28	19.49 ± 0.09 ^bA^	23.13 ± 0.37 ^aA^	−0.02 ± 0.01 ^aB^	359.99 ± 0.01 ^bA^	23.12 ± 0.36 ^aA^

Values sharing different lowercase letters in the table indicate a significant difference between each color parameter and storage time (*p* < 0.05). Values sharing different superscript uppercase letters in the table notify a significant difference between each color parameter and powder variant (*p* < 0.05).

**Table 3:** Phytochemical characteristics of added-value crackers and stability during 28 days of storage.

Phytochemicals /Time	Control		C1 with 1% Addition of V2		C2 with 3% Addition of V2	
	0	28 Days	0	28 Days	0	28 Days
TAC, μg C3G/g DW	-	-	27.57 ± 0.24 ^a^	22.24 ± 0.13 ^b^	39.18 ± 0.65 ^a^	33.51 ± 0.33 ^b^
TFC, mg QE/g DW	1.13 ± 0.02 ^a^	1.16 ± 0.01 ^a^	2.52 ±0.03 ^a^	2.49 ± 0.02 ^a^	4.57 ±0.19 ^a^	4.53 ± 0.01 ^a^
TPC, mg GAE/DW	1.47 ± 0.04 ^a^	1.50 ±0.01 ^a^	2.44 ±0.03 ^a^	2.38 ± 0.01 ^b^	3.62 ±0.01 ^a^	3.56 ± 0.01 ^b^
AA, mM TE/g DW	4.18 ± 0.11 ^a^	4.38 ± 0.07 ^a^	9.88 ± 0.06 ^a^	9.18 ± 0.08 ^b^	11.57 ± 0.06 ^a^	10.96 ± 0.03 ^b^

Different letters within the same row of each formulated cracker indicate a significant difference (*p*< 0.05).

## Data Availability

The data that support the findings of this study are available from the corresponding author (G.R.) upon reasonable request.
